# A Cheap and Simple Article of Nourishment for Infants

**Published:** 1851-02

**Authors:** 

**Affiliations:** Hamburg


*A cheap and simple article of Nourishment for Infants—Carrot Juice.* By Dr. Gumprecht of Hamburg.—Being impressed with the consideration that the nature of the artificial nourishment of young children deprived of the breast has a most important effect on their health, the author was anxious to find some aliment more supportable by the tender organism of infants than cow's milk and amylaceous matters, which mostly tend to acidity. An observation of Schmidtmann, that the pulp of carrots is a favorite article of diet for young children in Turkey, and is much used there, led him to turn his attention to this substance, which, as is well known, from the analyses of Wackenröder and Liebig, is particularly rich in albumen and sugar. It contains, however, also a quantity of ligneous fibres (cellulose), which is indigestible, and may therefore prove injurious.

He therefore thought of employing the thickened juice of carrots in the following way: An ounce of finely-rasped carrot pulp is mixed with two cups of cold *soft* water, and left for twelve hours, during which it is occasionaly stirred, then strained through a sieve, and the juice expressed from the pulp. This juice is then mixed with a sufficiency of bruised biscuit, or bruised crust of white bread, or a little arrowroot, a little sugar added, and the mixture heated, but not allowed to boil completely, so that the albuminous matter may not be coagulated. More sugar may be added if required.

The author considers the addition of biscuit or bread to be necessary, in order to furnish to the child all the requisite elements of nutrition—viz., albumen, starch, gluten, sugar, fat, and salts.

For sucklings deprived of the breast, the preparation is so far altered that the biscuit (1 part) is rubbed up with the carrot pulp (4 parts), then macerated and strained as above (we presume it is to be heated, though the author does not expressly say so.) To this juice the requisite amount of sugar, and a little salt are added, and the child fed with it from a sucking-bottle. Of course great cleanliness is requistie to prevent acidity being generated in the bottle, and for the same reason the juice, which readily undergoes fermentation, is to be kept in a very cool place, and prepared in small quantities at a time.

So far as the author's observation has gone, this system of nourishment has answered well, the children not only bearing it well, but taking the food readily, and he has received assurances of similar success from several of his professional brethren.

It is further stated by him that for older children the carrot pulp may be mixed with animal broth, and that large carrots answer better for the preparation of the juice than those which are young and small.

From the want of any direct experience of the above method, we cannot as yet express a judgment on the above proposition; but we must admit that the manufacture of the food does not appear (as the author seems to think) so easy that we can always reckon on its being successfully prepared. The chances of fermentation in such a juice are so great that it is to be feared it will hardly be avoided. We should regard any tendency to laxity of the bowels—so common an affection of children—as a complete contra-indication to its use. Further, there seems to be no reason why, even for young infants, some animal matter should not be added, in the form of broth or yolk of egg.—*Schmidt's Jahrbücher*, from *Journ. für Kinderkrankheiten*, July and August 1849.

In the *Journal für Kinderkrankheiten* for March and April, 1850, Dr. Gumprecht publishes letters from Dr. Müller, of Hamburg, Dr. Mauthner, of the Children's Hospital of Vienna, and Dr. Helmbrecht, of Brunswick, who state that they have followed his plan with satisfactory results. The last-named physician, however, remarks that if arrowroot is used to thicken the juice, it must be boiled, else the arrowroot will not dissolve, and this has the effect of coagulating the albuminous matter, which the author wishes to avoid. Dr. Helmbrecht suggests salep—a substance which is little used in this country, but which, we believe, is unjustly neglected.—*Monthly Journ. Med. Sci.*, Nov., 1850.

